# *Streptococcus pneumoniae* Modulates *Staphylococcus aureus* Biofilm Dispersion and the Transition from Colonization to Invasive Disease

**DOI:** 10.1128/mBio.02089-17

**Published:** 2018-01-09

**Authors:** Ryan M. Reddinger, Nicole R. Luke-Marshall, Shauna L. Sauberan, Anders P. Hakansson, Anthony A. Campagnari

**Affiliations:** a Department of Microbiology and Immunology, State University of New York at Buffalo, Buffalo, New York, USA; b Department of Translational Medicine, Lund University, Lund, Skåne, Sweden; University of Illinois at Chicago

**Keywords:** Polymicrobial infections, secondary bacterial pneumonia, *Staphylococcus aureus*, *Streptococcus pneumoniae*

## Abstract

*Streptococcus pneumoniae* and *Staphylococcus aureus* are ubiquitous upper respiratory opportunistic pathogens. Individually, these Gram-positive microbes are two of the most common causative agents of secondary bacterial pneumonia following influenza A virus infection, and they constitute a significant source of morbidity and mortality. Since the introduction of the pneumococcal conjugate vaccine, rates of cocolonization with both of these bacterial species have increased, despite the traditional view that they are antagonistic and mutually exclusive. The interactions between *S. pneumoniae* and *S. aureus* in the context of colonization and the transition to invasive disease have not been characterized. In this report, we show that *S. pneumoniae* and *S. aureus* form stable dual-species biofilms on epithelial cells *in vitro*. When these biofilms are exposed to physiological changes associated with viral infection, *S. pneumoniae* disperses from the biofilm, whereas *S. aureus* dispersal is inhibited. These findings were supported by results of an *in vivo* study in which we used a novel mouse cocolonization model. In these experiments, mice cocolonized in the nares with both bacterial species were subsequently infected with influenza A virus. The coinfected mice almost exclusively developed pneumococcal pneumonia. These results indicate that despite our previous report that *S. aureus* disseminates into the lungs of mice stably colonized with these bacteria following influenza A virus infection, cocolonization with *S. pneumoniae in vitro* and *in vivo* inhibits *S. aureus* dispersal and transition to disease. This study provides novel insight into both the interactions between *S. pneumoniae* and *S. aureus* during carriage and the transition from colonization to secondary bacterial pneumonia.

## IMPORTANCE

In this study, we demonstrate that *Streptococcus pneumoniae* can modulate the pathogenic potential of *Staphylococcus aureus* in a model of secondary bacterial pneumonia. We report that host physiological signals related to viral infection cease to elicit a dispersal response from *S. aureus* while in a dual-species setting with *S. pneumoniae*, in direct contrast to results of previous studies with each species individually. This study underscores the importance of studying polymicrobial communities and their implications in disease states.

## INTRODUCTION

*Streptococcus pneumoniae* (the pneumococcus), a Gram-positive diplococcus, is one of the most common opportunistic pathogens in the upper respiratory tract. By the age of 2 years, nearly 95% of children are colonized in the nasopharynx, in the form of a bacterial biofilm, by one of the greater than 90 serotypes of *S. pneumoniae*. Biofilm formation is a crucial step in pathogenesis, as biofilms promote bacterial persistence, competence, immune evasion, and resistance to antibiotics, all while serving as reservoirs for local and invasive disease (1–7). The pneumococcus, which predominantly causes upper respiratory tract infections, is the primary etiologic agent of both otitis media and secondary bacterial pneumonia (5, 8). Pneumococcal pneumonia following influenza A virus (IAV) infection poses a serious health threat to both young and elderly people. Recent studies have shown that host physiological changes in response to IAV infection, such as increased temperature, as well as the release of norepinephrine, glucose, and ATP, cause the pneumococcus to disperse from asymptomatically colonizing biofilms (9, 10). The bacteria, once dispersed from the biofilm in response to these stimuli, cause invasive disease, as they express an altered transcriptome and hypervirulent phenotype compared to the biofilm-associated bacteria (11–13).

Another common upper respiratory tract opportunistic pathogen is *Staphylococcus aureus*. *S. aureus* colonizes the anterior nares and nasopharynx of 30 to 80% of individuals, often in biofilms, which serve as a reservoir for local and invasive disease (14–17). In addition to many other diseases, *S. aureus* is a common cause of secondary bacterial pneumonia following IAV infection during seasonal influenza outbreaks and especially during pandemics (14, 18–25). Secondary staphylococcal pneumonia is severe, resulting in extensive damage to the respiratory tract, including necrosis, as well as dissemination into the bloodstream; it has been linked to mortality in previously healthy children with no underlying complications (21, 25–28). Recent studies from our laboratory have demonstrated that physiological changes linked to IAV infection induce *S. aureus* to disperse from biofilms *in vitro* and to disperse and disseminate from asymptomatically colonized murine nasal tissues into the lungs, where they cause pronounced staphylococcal secondary pneumonia (29).

The human nasopharynx is a very diverse physiological niche, and the microbiome has become much more complex since the introduction of the pneumococcal conjugate vaccine (PCV) (15, 30–32). While this vaccine has been very effective against relevant invasive pneumococcal serotypes, non-PCV serotype replacement has occurred and may be a contributing factor for the rise of *S. aureus* colonization of the nasopharynx, otitis media, and staphylococcal secondary pneumonia (22, 23, 25, 33, 34). It is possible that the change in colonizing pneumococcal serotypes in the nasopharynx has altered the competitive environment of this niche, facilitating the emergence of *S. aureus*. Previous *in vitro* studies have suggested that these species are antagonistic, whereby the pneumococcus uses hydrogen peroxide production to kill *S. aureus* (35). In contrast, subsequent models of cocolonization with both species have shown that this antagonistic relationship does not occur in animal models (36, 37). Moreover, this observation has been supported by recent epidemiological studies reporting an increased cocolonization rate of the human nasopharynx, as both species were detected in 10 to 24% of patients sampled (38–40).

The complex nature of the interaction between *S. aureus* and *S. pneumoniae* in the nasopharynx and the clinical increase in cocolonization warrants further analyses. No studies to date have examined the dynamics of dual-species cocolonization or the transition from colonization to secondary bacterial pneumonia. Therefore, we have developed an *in vitro* and *in vivo* system to investigate the effects on dual-species biofilms of host physiological changes related to IAV infection. Our results suggest that while stable dual-species biofilms can be formed and stable cocolonization of the nasal tissue can be established, the presence of *S. pneumoniae* inhibits staphylococcal dispersal and dissemination to invasive disease while remaining unaffected itself.

## RESULTS

### *S. aureus* and *S. pneumoniae* form robust dual-species biofilms *in vitro* on fixed H292 cells.

**FIG 1:**
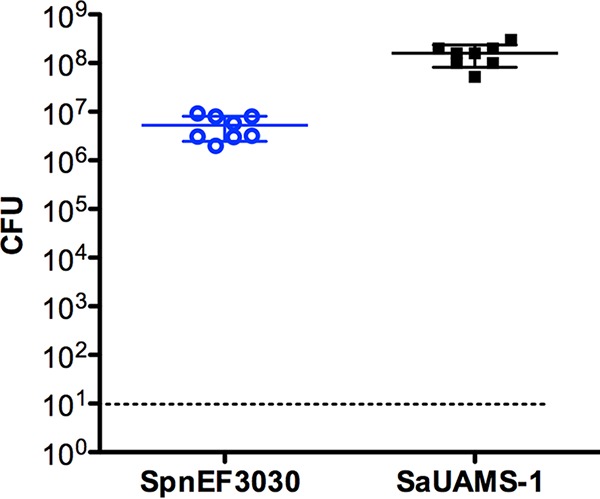
*S. pneumoniae* EF3030 and *S. aureus* UAMS-1 form dual-species biofilms *in vitro*. Biofilm-associated EF3030 and UAMS-1 were enumerated from cocultured biofilms after 48 h (*n* = 8). Both species of bacteria were recovered from each well. The dashed line indicates the limit of detection. Solid bars are means ± standard deviations.

**FIG 2:**
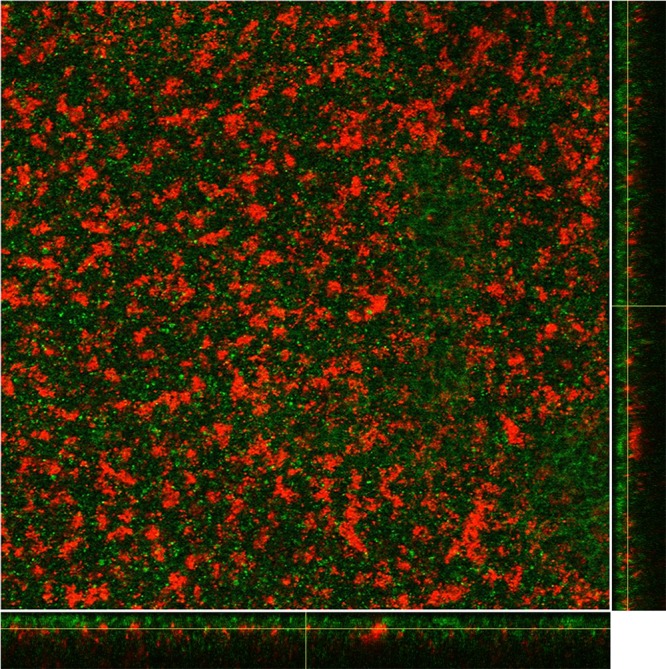
CLSM images of dual-species biofilms formed by *S. pneumoniae* EF3030-GFP (green) and *S. aureus* UAMS1-dsRED (red) following 48 h of coculture. The image shows the average intensity projections through the confocal image stack, with the maximum-intensity *x*-*z* and *y*-*z* projections shown along the bottom and side of the image; a representative image of a single slice from the central region of the biofilm is shown. Images were produced using ImageJ.

Previous studies have shown that both *S. aureus* strain UAMS-1 and *S. pneumoniae* strain EF3030 form robust single-species biofilms in our *in vitro* model system, and these closely mimic *in vivo* biofilms (29, 41). Given the rise in cocolonization rates with these species since introduction of the PCV, we performed studies to determine if these two species form biofilms on fixed human H292 cells *in vitro*. The data in Fig. 1 indicate that, following coculture using this *in vitro* system, high levels of both UAMS-1 and EF3030 were recovered from harvested biofilms (approximately 10^8^ and 10^7^ CFU per well, respectively). These quantitative data suggest that in a dual-species model, *S. aureus* UAMS-1 and *S. pneumoniae* EF3030 form stable biofilms for 48 h under conditions that more closely mimic the microenvironment *in vivo*. A representative dual-species biofilm was visualized via confocal laser scanning microscopy (CLSM). In Fig. 2, both bacterial strains are shown colocalized in the mixed-species biofilm, with towers of UAMS-1 visible over a mat-like distribution of EF3030.

### The *S. aureus* response to host physiological changes induced by viral infection is modulated in the presence of *S. pneumoniae in vitro*.

**FIG 3:**
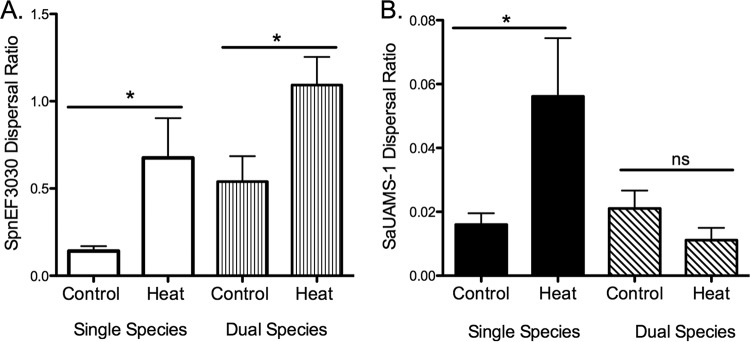
*In vitro* dispersal of 48 h *S. pneumoniae* EF3030 (A) and *S. aureus* UAMS-1 (B) single-species and dual-species biofilms following 4 h of heat treatment (38.5°C). Dispersal values are presented as the ratio of supernatant to biofilm from a minimum of four independent experiments (means ± standard errors of the means). Statistical analysis of the effect heat dispersal compared to controls was performed with the unpaired Student *t* test. *, *P* < 0.05.

As mentioned above, IAV infection induces a wide range of host physiological responses. We have previously shown that both *S. pneumoniae* and *S. aureus* in single-species studies disperse from the biofilm in response to physiological changes in the host that are associated with IAV infection *in vitro* and disseminate to the lungs *in vivo* (9, 29). In these previous studies, the greatest effect by a single factor for both organisms was seen upon exposure to febrile-range temperatures. We repeated this experiment to determine if this same physiological change induced dispersal in a dual-species biofilm. Figure 3A shows that *S. pneumoniae* EF3030 responded to heat stimuli by dispersing from the biofilm, as previously reported (9). However, in contrast to our recent data (29), *S. aureus* UAMS-1 did not disperse from the dual-species biofilm in response to this stimulus, as shown in Fig. 3B. Moreover, staphylococcal dispersal inhibition was also observed when *S. pneumoniae* EF3030 was cocultured with *S. aureus* NRS123, and also coculture of *S. aureus* NRS123 in the presence of *S. pneumoniae* D39 (data not shown). These data demonstrate stable dual-species biofilm formation and dispersal inhibition are not strain specific. Taken together, these *in vitro* studies suggest that pneumococci may modulate the response of staphylococci to physiological changes associated with IAV infection.

### *Streptococcus mitis* does not inhibit *S. aureus* dispersal.

**FIG 4:**
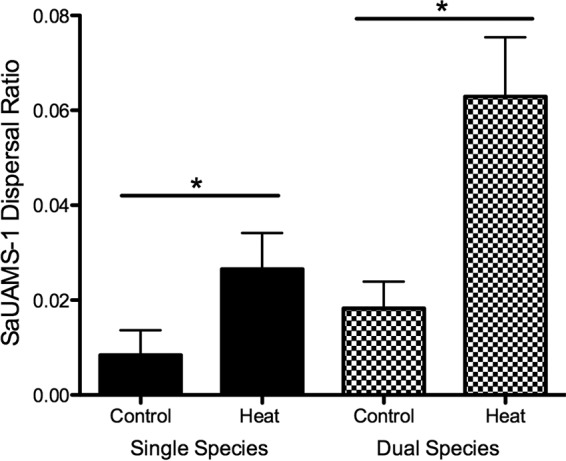
*In vitro* dispersal of *S. aureus* UAMS-1 from 48-h single-species biofilms compared to dual-species biofilms with *S. mitis* NS5S following 4 h of heat treatment (38.5°C). Dispersal values are presented as the ratio of supernatant to biofilm from a minimum of four independent experiments (mean result ± standard deviation). Statistical analysis on heat dispersal versus that in controls was performed with the unpaired Student *t* test. *, *P* < 0.05.

We next determined if *S. aureus* dispersal could be inhibited by another streptococcal species. *S. mitis* was selected for these experiments, as this organism is a closely genetically related oropharyngeal commensal that shares physiological and molecular traits with *S. pneumoniae*, in addition to colonizing the same human niche. We grew dual-species biofilms of *S. aureus* UAMS-1 and *S. mitis* strain NS51, an ATCC type strain shown in previous studies to form biofilms (42). Figure 4 shows that *S. aureus* UAMS-1 dispersal was unaffected by the presence of *S. mitis*, suggesting that the previously noted inhibition may be pneumococcus specific.

### *S. aureus* and *S. pneumoniae* asymptomatically cocolonize the murine nasopharynx.

**FIG 5:**
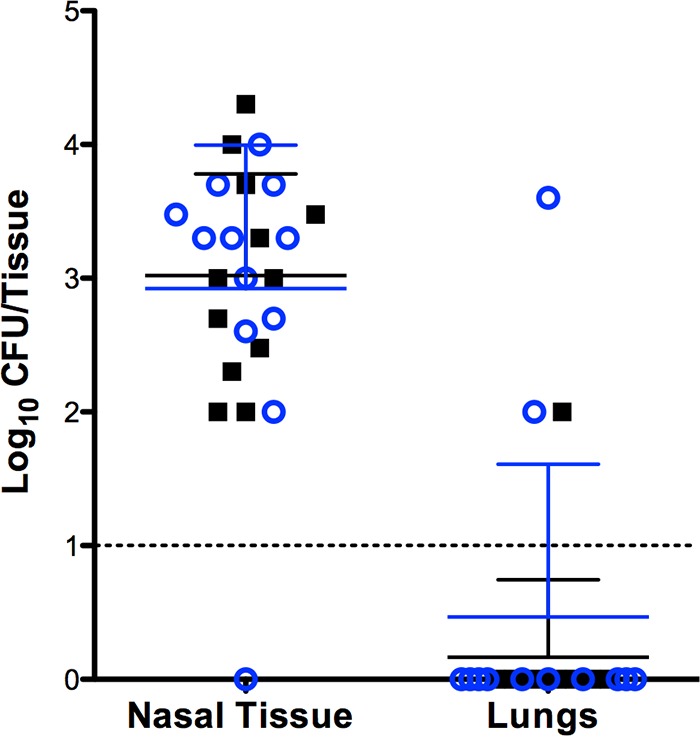
Dual-species colonization of the murine nasal mucosa. Samples were harvested 48 h after colonization; each point presents the CFU recovered from individual mice (*n* = 12). Blue circles represent *S. pneumoniae* EF3030, and the black squares represent *S. aureus* UAMS-1. The dashed line indicates the limit of detection. Bars indicate standard deviations.

A majority of murine secondary bacterial pneumonia studies consist of an aspiration of IAV followed 3 to 7 days later with a second aspiration of a high titer of bacteria. As the bacteria are introduced directly into the lungs, these experimental *in vivo* systems bypass the initial colonization of the nasal tissues, which is likely important for infection. Based on our *in vitro* biofilm results, we performed studies using our recently described murine nasal colonization model to determine if *S. aureus* UAMS-1 and *S. pneumoniae* EF3030 form dual-species biofilms *in vivo*. The data in Fig. 5 show that nasal tissues harvested from the inoculated mice were cocolonized with both UAMS-1 and EF3030, suggesting that these two species can concurrently and stably colonize the murine nasopharynx for 48 h. Moreover, it is important to note that bacteria remained in the nasopharynx in the majority of these animals, and the few mice form which bacteria were recovered from the lungs did not exhibit any detectable symptoms of pneumonia.

### IAV coinfection in asymptomatically cocolonized mice primarily leads to secondary pneumococcal pneumonia.

**FIG 6:**
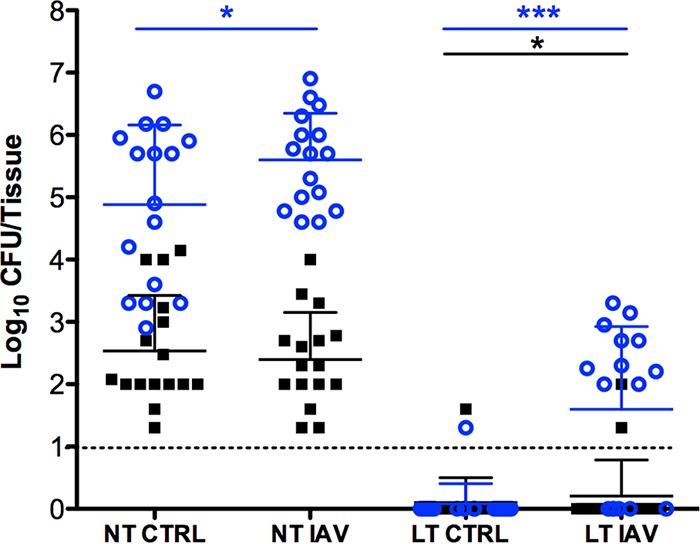
Dissemination following IAV coinfection. Forty-eight hours post-bacterial colonization, IAV cohorts received intranasal virus inoculations while control cohorts (CTRL) received only saline, and the infection was allowed to progress an additional 48 h prior to harvest. Each point represents the CFU recovered from nasal tissues (NT) and lung tissues (LT) harvested from individual mice (*n* = 16) 96 h after cocolonization with *S. pneumoniae* EF3030 (blue circles) and *S. aureus* UAMS-1 (black squares). The dashed line indicates the limit of CFU detection. Solid bars indicate the standard deviations. Statistical analysis was performed with the nonparametric Mann-Whitney test. *, *P* < 0.05; ***, *P* < 0.001.

Based on the *in vitro* dispersal data, we performed the following studies to determine if *S. pneumoniae* also modulated *S. aureus* dispersal *in vivo*. Mice were cocolonized with *S. aureus* UAMS-1 and *S. pneumoniae* EF3030 for 48 h and then infected with IAV strain PR8 via aspiration. At 48 h post-virus infection, animals were sacrificed and harvested nasal and lung tissues were evaluated for bacterial burden by CFU counts. Figure 6 shows that 96 h after initial bacterial inoculations, comparable bacterial burdens of *S. aureus* and *S. pneumoniae* were detected in murine nasal tissues for both the control cohort and the cohort that received both bacteria and virus. Only mice that received both virus and bacteria, however, developed pronounced secondary bacterial pneumonia, displaying piloerection, lethargy, and increased body temperature. Moreover, these data indicate that the majority of these mice (62.5%) developed secondary pneumococcal pneumonia, while only two mice had staphylococci in the lungs (12.5%). In addition, none of the animals had both bacterial species recovered from the lungs; in all cases, the pneumonia was monomicrobial. These results suggest the effect on dispersal that *S. pneumoniae* exerts on *S. aureus in vitro* may also be occurring *in vivo*, thereby inhibiting *S. aureus* from disseminating to the lungs when cocolonizing pneumococci are present in the upper respiratory tract.

## DISCUSSION

While IAV causes significant amounts of tissue damage and inflammation, the development of secondary bacterial pneumonia is one of the most serious complications associated with infection. It is a primary cause of both morbidity and mortality during seasonal influenza epidemics and during influenza pandemics (22, 23, 43–45). The two main etiologic agents of secondary bacterial pneumonia are the upper respiratory pathogens *S. pneumoniae* and *S. aureus*. It appears that biofilms colonizing the upper respiratory tract are the most likely reservoir for invasive disease caused by both of these species. Interestingly, epidemiological studies have shown an increase in the incidence of cocolonization in the nasopharynx by both *S. pneumoniae* and *S. aureus* even though these species are traditionally viewed as mutually exclusive and antagonistic species (35, 38–40).

Despite this increase in cocolonization of the nasopharynx, there are essentially no case reports of dual-species secondary bacterial pneumonia. This suggests that although *S. pneumoniae* and *S. aureus* can coexist on the mucosal surface of the upper respiratory tract and both species are able to cause secondary bacterial pneumonia, the virulence capacity of *S. aureus* appears to be diminished in the presence of the pneumococcus. Despite this intriguing observation, few studies have been performed to investigate these complex bacterial interactions in the context of secondary bacterial pneumonia. Furthermore, little is known about the events involved in the transition from colonization to invasive disease or about the effects of a polymicrobial environment on this transition.

We began to address this gap in knowledge by investigating the transition of *S. pneumoniae* and *S. aureus* from colonization to invasive disease following IAV infection in a dual-species model. We previously showed that, individually, both bacterial species will respond to physiological changes induced by IAV infection by dispersing from the biofilm and disseminating from the nasal tissue to the lungs (9, 29). Using a novel *in vitro* dual-species biofilm model, we were able to demonstrate that *S. pneumoniae* and *S. aureus* form robust dual-species biofilms on prefixed upper respiratory epithelial cells that remain stable for up to 48 h. Biofilm formation at 34°C with the addition of an epithelial cell substratum more closely mimics the ecologic niche of the upper respiratory tract (41). These findings suggest that the previously reported antagonistic relationship between these species may be the result of various environmental factors, including growth conditions, which could affect the ability of these two bacterial species to coexist.

Using this physiologically relevant model, we were able to investigate the effects of host changes in response to IAV infection on dual-species biofilms. Our results demonstrated that in a dual-species biofilm, *S. pneumoniae* responds to these stimuli by dispersing from the biofilm, consistent with previous reports using single-species biofilms (9). However, *S. aureus* did not disperse from the dual-species biofilm, which is in direct contrast to our recent report showing that *S. aureus* disperses from a single-species biofilm in response to the same stimuli (29). Importantly, *S. aureus* dispersal was not inhibited in the presence of *S. mitis*, an oropharyngeal colonizer that is closely related to *S. pneumoniae*; these findings support the specificity of the observed inhibition. To our knowledge, these are the first results suggesting that *S. pneumoniae* modulates the pathogenesis of *S. aureus*, although the mechanism responsible for this observation remains currently undefined and warrants additional studies.

Our *in vitro* dual-species biofilm results were confirmed *in vivo*. Using an intranasal colonization model, we demonstrated that mice could be simultaneously colonized with both *S. pneumoniae* and *S. aureus* for up to 96 h. These results extend two previous studies reporting dual colonization in a neonatal mouse and rat nasal model for 72 h (36, 37). We investigated the effect of concomitant IAV infection on cocolonized mice. Previously published models of secondary bacterial pneumonia have overlooked the natural route of infection, bypassing the transition from colonization to invasive disease. Our novel secondary pneumonia model allows us to investigate this critical step in pathogenesis, and our *in vivo* coinfection experiments confirmed the *in vitro* dispersal results. When cocolonized mice receive IAV, the majority of these mice develop pneumococcal pneumonia within 48 h of viral infection. The finding that few of these mice developed staphylococcal pneumonia suggested that the inhibition of biofilm dispersal that we observed *in vitro* is a phenomenon that also occurs *in vivo*. Furthermore, these data show that the nasal tissues of the mice remain stably colonized with both species for up to 96 h. These are the first studies to effectively demonstrate that colonization with these two species of bacteria alters the pathogenesis of one species in a host environment. It is important to note that in all mice that developed pneumonia, the bacteria harvested from the infected lung tissue were exclusively pneumococci or staphylococci, but never both. The disseminated lung infections were strictly monomicrobial, even though the nasal tissues indicated stable cocolonization by both species. The finding that none of the cocolonized IAV-infected mice developed a dual-species bacterial pneumonia is consistent with most clinical case reports of secondary bacterial pneumonia (46).

There are numerous potential explanations for these results. A recent report showed that the transcriptional profile of *S. pneumoniae* shifts in response to dispersal from a stable biofilm, with an upregulation of pneumococcal virulence factors and bacteriocins (11). It is possible that this increase in bacteriocin production by dispersing streptococci leads to the killing of *S. aureus* as it also disperses from the biofilm, a common method used to eliminate competition (47–50). Another explanation is that *S. pneumoniae* biofilm structural components trap *S. aureus* in the extracellular matrix, preventing dispersal. Alterations in the regulation of known *S. aureus* biofilm dispersal mechanisms, including exoenzymes and surfactants, such as proteases, nucleases, and phenol-soluble modulins, via cocolonization with *S. pneumoniae* could negatively affect *S. aureus* dispersal from established biofilms (recently reviewed in references 51 and 52). Alternatively, the presence of the pneumococcus may induce *S. aureus* to increase expression of adhesins and/or other biofilm-associated factors, thus promoting a more cohesive structure that stunts dispersal. These possibilities are merely speculative, and detailed mechanistic and transcriptomic studies designed to delineate these complex bacterial interactions are warranted prior to making any definitive conclusions.

This study is the first to demonstrate that the presence of *S. pneumoniae* alters the pathogenesis of *S. aureus*. These findings may provide insight into the rise of *S. aureus* secondary bacterial pneumonia since the introduction of the PCV. While global use of PCV has greatly reduced carriage of vaccine serotype pneumococci and invasive disease caused by these strains, colonization by non-PCV serotype replacement strains continues to occur, and broad PCV implementation has clearly changed the microbiome of the nasopharynx overall. More studies are needed to better understand the complex interactions that exist between various bacterial species that share common microenvironments and the contributions of interspecies competition to the dynamics of the microbiome of a given ecologic niche. Our data provide the foundation for future experiments designed to delineate the mechanisms used by *S. pneumoniae* and *S. aureus* during cocolonization of the human upper respiratory tract and define their contributions to the subsequent development of invasive disease.

## MATERIALS AND METHODS

### Reagents.

Bacterial and cell culture materials were from VWR (Radnor, PA) and Invitrogen (Carlsbad, CA). Chemically defined pneumococcal growth medium (CDM) was formulated as previously described (53). Sheep blood was purchased from Biolink, Liverpool, NY.

### Eukaryotic cell lines and bacterial and viral strains.

NCI-H292 bronchial epithelial cells were grown and prepared as previously described (54). This study used *Streptococcus pneumoniae* strains EF3030, a serotype 19F otitis media isolate (55), and D39, a classical serotype 2 Avery strain (56). *Staphylococcus aureus* strain UAMS-1 (ATCC 49230) (57) was a generous gift from Steve Gill (University of Rochester, Rochester, NY), and methicillin-resistant strain NRS123 was from the Network on Antimicrobial Resistance in *Staphylococcus aureus* (NARSA) (58). *Streptococcus mitis* strain NS51 was obtained from ATCC (ATCC 49456) (42). Chromosomally encoded fluorescent variants for confocal microscopy were generated in *S. pneumoniae* EF3030 by natural transformation with the *hlpA-gfp_Camr* construct from strain JVW500, as described elsewhere (59) (strain JVW500 was a generous gift from Jan-Willem Veening, University of Lausanne, Lausanne, Switzerland), creating strain EF3030-GFP. UAMS-1–dsRED, was constructed via chromosomal integration using pRFP-F, as described previously (60), and was a generous gift from Steve Gill (University of Rochester, Rochester, NY). Bacteria were grown in CDM at 37°C unless otherwise noted. Viral stocks of influenza A virus strain A/PR/8/34 were a mouse-adapted H1N1 strain (with titers determined), generously provided by Paul Knight (University at Buffalo, Buffalo, NY) and used for the viral coinfection studies (61).

### Static biofilms on fixed epithelial substratum.

Static pneumococcal biofilms were grown as previously described (41). *S. aureus* biofilms were grown using the same model with slight modifications. Briefly, *S. aureus* was grown in CDM at 37°C to mid-log phase. Bacteria were diluted to a final inoculum of 2 × 10^7^ CFU in a 500-μl volume of CDM and seeded onto a fixed H292 cell substratum with 500 μl of additional CDM. Biofilms were placed at 34°C for growth, with medium changed every 12 h for 48 h. Dual-species biofilms were grown using the same model with additional modifications. In brief, streptococci were grown in CDM to mid-log phase, diluted in 500 μl of fresh medium to a final inoculum of 5 × 10^6^ CFU, and seeded onto fixed H292 epithelial cells in an additional 500 μl of fresh medium. Biofilms were placed at 34°C for 24 h. Five hundred microliters of CDM was removed, and 2 × 10^7^ CFU of *S. aureus* grown as mentioned above were added gently to the preformed streptococcal biofilms and then placed at 34°C for an additional 24 h. A minimum of three independent assays with four replicates each were performed.

### Biofilm dispersal.

Static monomicrobial or dual-species biofilms were prepared as outlined above. Biofilms were dispersed as previously described (9). Briefly, biofilms were shifted to a febrile-range temperature of 38.5°C for 4 h. Supernatants from each biofilm were removed, sonicated, and plated onto blood agar plates for bacterial quantification. Remaining biofilm bacteria were suspended in phosphate-buffered saline, sonicated, and plated onto blood agar plates for bacterial quantification. Values reported are the calculated ratios of supernatant to biofilm CFU, a measure of bacterial dispersal, as previously reported (29).

### Confocal microscopy.

In order to identify and localize individual bacteria in the dual-species biofilms, cocolonized biofilms were prepared on a fixed epithelial substratum as described above, except we used glass-bottom 24-well plates (MatTek Corp., Ashland, MA) and chromosomally encoded fluorescent constructs (EF3030-GFP [green] and UAMS-1–dsRED [red]). Image stacks were acquired by CLSM at 630× magnification on a Zeiss Axiovert 200 M inverted microscope with an attached Zeiss LSM 510 Meta NLO imaging system. Images were produced from the raw LSM files by using the freely available ImageJ software analysis program.

### Mouse nasopharyngeal colonization and influenza virus infection model.

Nasopharyngeal single- and dual-species colonization experiments were performed as previously described, with limited modifications (62). Briefly, nonanesthetized 5-week-old BALB/cByJ mice were intranasally inoculated with either 2 × 10^8^ CFU of *S. aureus* UAMS-1 or 5 × 10^6^ CFU of *S. pneumoniae* EF3030 in a 20-μl volume by pipetting the inoculum into the nares. For dual-species colonization experiments, nonanesthetized 5-week-old BALB/cByJ mice were intranasally inoculated with both UAMS-1 and EF3030 in a 20-μl volume as described above with 30 min in between inoculations. For influenza virus coinfection experiments, mice colonized for a period of 48 h were anesthetized with isofluorane and 100 PFU of IAV in 20 μl was pipetted into the nares. At 48 h post-viral inoculation (96 h post-bacterial colonization), nasopharyngeal tissue and lungs were collected, homogenized, and plated onto blood agar plates to assess bacterial burdens.

### Statistical analyses.

Statistical significance was determined using the Mann-Whitney U test or Student’s *t* test, as indicated, and Prism 5 from GraphPad Software, Inc. (La Jolla, CA). A *P* value of <0.05 was considered significant.

## References

[1] Bogaert D, De Groot R, Hermans PW 2004 Streptococcus pneumoniae colonisation: the key to pneumococcal disease. Lancet Infect Dis 4:144–154. doi:10.1016/S1473-3099(04)00938-7. 14998500

[2] Gray BM, Converse GM III, Dillon HC Jr 1980 Epidemiologic studies of Streptococcus pneumoniae in infants: acquisition, carriage, and infection during the first 24 months of life. J Infect Dis 142:923–933. doi:10.1093/infdis/142.6.923. 7462701

[3] Huebner RE, Wasas AD, Klugman KP, Paediatric Study Group 2000 Prevalence of nasopharyngeal antibiotic-resistant pneumococcal carriage in children attending private paediatric practices in Johannesburg. S Afr Med J 90:1116–1121. 11196033

[4] Huebner RE, Dagan R, Porath N, Wasas AD, Klugman KP 2000 Lack of utility of serotyping multiple colonies for detection of simultaneous nasopharyngeal carriage of different pneumococcal serotypes. Pediatr Infect Dis J 19:1017–1020. doi:10.1097/00006454-200010000-00019. 11055610

[5] Charalambous BM, Leung MH 2012 Pneumococcal sepsis and nasopharyngeal carriage. Curr Opin Pulm Med 18:222–227. doi:10.1097/MCP.0b013e328352103b. 22343427

[6] Marks LR, Reddinger RM, Hakansson AP 2012 High levels of genetic recombination during nasopharyngeal carriage and biofilm formation in Streptococcus pneumoniae. mBio 3:e0200-12. doi:10.1128/mBio.00200-12. PMC3448161 23015736

[7] Marks LR, Reddinger RM, Hakansson AP 2014 Biofilm formation enhances fomite survival of Streptococcus pneumoniae and Streptococcus pyogenes. Infect Immun 82:1141–1146. doi:10.1128/IAI.01310-13. 24371220 PMC3957990

[8] Vernatter J, Pirofski LA 2013 Current concepts in host-microbe interaction leading to pneumococcal pneumonia. Curr Opin Infect Dis 26:277–283. doi:10.1097/QCO.0b013e3283608419. 23571695 PMC4237063

[9] Marks LR, Davidson BA, Knight PR, Hakansson AP 2013 Interkingdom signaling induces Streptococcus pneumoniae biofilm dispersion and transition from asymptomatic colonization to disease. mBio 4:e00438-13. doi:10.1128/mBio.00438-13. 23882016 PMC3735180

[10] Chao Y, Marks LR, Pettigrew MM, Hakansson AP 2014 Streptococcus pneumoniae biofilm formation and dispersion during colonization and disease. Front Cell Infect Microbiol 4:194. doi:10.3389/fcimb.2014.00194. 25629011 PMC4292784

[11] Pettigrew MM, Marks LR, Kong Y, Gent JF, Roche-Hakansson H, Hakansson AP 2014 Dynamic changes in the Streptococcus pneumoniae transcriptome during transition from biofilm formation to invasive disease upon influenza A virus infection. Infect Immun 82:4607–4619. doi:10.1128/IAI.02225-14. 25135685 PMC4249342

[12] Sanchez CJ, Kumar N, Lizcano A, Shivshankar P, Dunning Hotopp JC, Jorgensen JH, Tettelin H, Orihuela CJ 2011 Streptococcus pneumoniae in biofilms are unable to cause invasive disease due to altered virulence determinant production. PLoS One 6:e28738. doi:10.1371/journal.pone.0028738. 22174882 PMC3234282

[13] Sanchez CJ, Hurtgen BJ, Lizcano A, Shivshankar P, Cole GT, Orihuela CJ 2011 Biofilm and planktonic pneumococci demonstrate disparate immunoreactivity to human convalescent sera. BMC Microbiol 11:245. doi:10.1186/1471-2180-11-245. 22047041 PMC3216281

[14] van den Bergh MR, Biesbroek G, Rossen JW, de Steenhuijsen Piters WA, Bosch AA, van Gils EJ, Wang X, Boonacker CW, Veenhoven RH, Bruin JP, Bogaert D, Sanders EA 2012 Associations between pathogens in the upper respiratory tract of young children: interplay between viruses and bacteria. PLoS One 7:e47711. doi:10.1371/journal.pone.0047711. 23082199 PMC3474735

[15] Bosch AA, Biesbroek G, Trzcinski K, Sanders EA, Bogaert D 2013 Viral and bacterial interactions in the upper respiratory tract. PLoS Pathog 9:e1003057. doi:10.1371/journal.ppat.1003057. 23326226 PMC3542149

[16] Wertheim HF, Vos MC, Ott A, van Belkum A, Voss A, Kluytmans JA, van Keulen PH, Vandenbroucke-Grauls CM, Meester MH, Verbrugh HA 2004 Risk and outcome of nosocomial Staphylococcus aureus bacteraemia in nasal carriers versus non-carriers. Lancet 364:703–705. doi:10.1016/S0140-6736(04)16897-9. 15325835

[17] Esposito S, Terranova L, Zampiero A, Ierardi V, Rios WP, Pelucchi C, Principi N 2014 Oropharyngeal and nasal Staphylococcus aureus carriage by healthy children. BMC Infect Dis 14:723. doi:10.1186/s12879-014-0723-9. 25551464 PMC4299802

[18] Shieh WJ, Blau DM, Denison AM, Deleon-Carnes M, Adem P, Bhatnagar J, Sumner J, Liu L, Patel M, Batten B, Greer P, Jones T, Smith C, Bartlett J, Montague J, White E, Rollin D, Gao R, Seales C, Jost H, Metcalfe M, Goldsmith CS, Humphrey C, Schmitz A, Drew C, Paddock C, Uyeki TM, Zaki SR 2010 2009 Pandemic influenza A(H1N1): pathology and pathogenesis of 100 fatal cases in the United States. Am J Pathol 177:166–175. doi:10.2353/ajpath.2010.100115. 20508031 PMC2893660

[19] Punpanich W, Chotpitayasunondh T 2012 A review on the clinical spectrum and natural history of human influenza. Int J Infect Dis 16:e714–e723. doi:10.1016/j.ijid.2012.05.1025. 22784546

[20] Murray RJ, Robinson JO, White JN, Hughes F, Coombs GW, Pearson JC, Tan HL, Chidlow G, Williams S, Christiansen KJ, Smith DW 2010 Community-acquired pneumonia due to pandemic A(H1N1)2009 influenza virus and methicillin resistant Staphylococcus aureus co-infection. PLoS One 5:e8705. doi:10.1371/journal.pone.0008705. 20090931 PMC2806836

[21] Bhat N, Wright JG, Broder KR, Murray EL, Greenberg ME, Glover MJ, Likos AM, Posey DL, Klimov A, Lindstrom SE, Balish A, Medina MJ, Wallis TR, Guarner J, Paddock CD, Shieh WJ, Zaki SR, Sejvar JJ, Shay DK, Harper SA, Cox NJ, Fukuda K, Uyeki TM, Influenza Special Investigations Team 2005 Influenza-associated deaths among children in the United States, 2003–2004. N Engl J Med 353:2559–2567. doi:10.1056/NEJMoa051721. 16354892

[22] Dawood FS, Fiore A, Kamimoto L, Nowell M, Reingold A, Gershman K, Meek J, Hadler J, Arnold KE, Ryan P, Lynfield R, Morin C, Baumbach J, Zansky S, Bennett NM, Thomas A, Schaffner W, Kirschke D, Finelli L, Emerging Infections Program (EIP) Network 2010 Influenza-associated pneumonia in children hospitalized with laboratory-confirmed influenza, 2003–2008. Pediatr Infect Dis J 29:585–590. doi:10.1097/INF.0b013e3181d411c5. 20589966 PMC5856105

[23] Dawood FS, Chaves SS, Pérez A, Reingold A, Meek J, Farley MM, Ryan P, Lynfield R, Morin C, Baumbach J, Bennett NM, Zansky S, Thomas A, Lindegren ML, Schaffner W, Finelli L, Emerging Infections Program Network 2014 Complications and associated bacterial coinfections among children hospitalized with seasonal or pandemic influenza, United States, 2003–2010. J Infect Dis 209:686–694. doi:10.1093/infdis/jit473. 23986545

[24] Rice TW, Rubinson L, Uyeki TM, Vaughn FL, John BB, Miller RR III, Higgs E, Randolph AG, Smoot BE, Thompson BT, NHLBI ARDS Network 2012 Critical illness from 2009 pandemic influenza A virus and bacterial coinfection in the United States. Crit Care Med 40:1487–1498. doi:10.1097/CCM.0b013e3182416f23. 22511131 PMC3653183

[25] Randolph AG, Vaughn F, Sullivan R, Rubinson L, Thompson BT, Yoon G, Smoot E, Rice TW, Loftis LL, Helfaer M, Doctor A, Paden M, Flori H, Babbitt C, Graciano AL, Gedeit R, Sanders RC, Giuliano JS, Zimmerman J, Uyeki TM, Pediatric Acute Lung Injury and Sepsis Investigator's Network, National Heart, Lung, and Blood Institute ARDS Clinical Trials Network 2011 Critically ill children during the 2009–2010 influenza pandemic in the United States. Pediatrics 128:e1450–e1458. doi:10.1542/peds.2011-0774. 22065262 PMC3387899

[26] Park SS, Kim SH, Kim M, Kim JW, Ko YM, Kim SK, Kim SH, Kim CH 2015 A case of severe pseudomembranous tracheobronchitis complicated by co-infection of influenza A (H1N1) and Staphylococcus aureus in an immunocompetent patient. Tuberc Respir Dis 78:366–370. doi:10.4046/trd.2015.78.4.366. PMC4620332 26508926

[27] Reed C, Kallen AJ, Patton M, Arnold KE, Farley MM, Hageman J, Finelli L 2009 Infection with community onset Staphylococcus aureus and influenza virus in hospitalized children. Pediatr Infect Dis J 28:572–576. doi:10.1097/INF.0b013e31819d8b71. 19478685

[28] Kallen AJ, Reed C, Patton M, Arnold KE, Finelli L, Hageman J 2010 Staphylococcus aureus community onset pneumonia in patients admitted to children’s hospitals during autumn and winter of 2006–2007. Epidemiol Infect 138:666–672. doi:10.1017/S095026880999135X. 19961644

[29] Reddinger RM, Luke-Marshall NR, Hakansson AP, Campagnari AA 2016 Host physiologic changes induced by influenza A virus lead to Staphylococcus aureus biofilm dispersion and transition from asymptomatic colonization to invasive disease. mBio 7:e01235-16. doi:10.1128/mBio.01235-16. 27507829 PMC4981728

[30] Bogaert D, Keijser B, Huse S, Rossen J, Veenhoven R, van Gils E, Bruin J, Montijn R, Bonten M, Sanders E 2011 Variability and diversity of nasopharyngeal microbiota in children: a metagenomic analysis. PLoS One 6:e17035. doi:10.1371/journal.pone.0017035. 21386965 PMC3046172

[31] Pelton SI 2012 Regulation of bacterial trafficking in the nasopharynx. Paediatr Respir Rev 13:150–153. doi:10.1016/j.prrv.2012.04.001. 22726870 PMC3383606

[32] Odutola A, Antonio M, Owolabi O, Bojang A, Foster-Nyarko E, Donkor S, Adetifa I, Taylor S, Bottomley C, Greenwood B, Ota M 2013 Comparison of the prevalence of common bacterial pathogens in the oropharynx and nasopharynx of Gambian infants. PLoS One 8:e75558. doi:10.1371/journal.pone.0075558. 24086570 PMC3781055

[33] Spijkerman J, Prevaes SM, van Gils EJ, Veenhoven RH, Bruin JP, Bogaert D, Wijmenga-Monsuur AJ, van den Dobbelsteen GP, Sanders EA 2012 Long-term effects of pneumococcal conjugate vaccine on nasopharyngeal carriage of S. pneumoniae, S. aureus, H. influenzae and M. catarrhalis. PLoS One 7:e39730. doi:10.1371/journal.pone.0039730. 22761879 PMC3382588

[34] Lee MH, Arrecubieta C, Martin FJ, Prince A, Borczuk AC, Lowy FD 2010 A postinfluenza model of Staphylococcus aureus pneumonia. J Infect Dis 201:508–515. doi:10.1086/650204. 20078212 PMC3664424

[35] Regev-Yochay G, Trzcinski K, Thompson CM, Malley R, Lipsitch M 2006 Interference between Streptococcus pneumoniae and Staphylococcus aureus: in vitro hydrogen peroxide-mediated killing by Streptococcus pneumoniae. J Bacteriol 188:4996–5001. doi:10.1128/JB.00317-06. 16788209 PMC1482988

[36] Park B, Nizet V, Liu GY 2008 Role of Staphylococcus aureus catalase in niche competition against Streptococcus pneumoniae. J Bacteriol 190:2275–2278. doi:10.1128/JB.00006-08. 18223076 PMC2293205

[37] Margolis E 2009 Hydrogen peroxide-mediated interference competition by Streptococcus pneumoniae has no significant effect on Staphylococcus aureus nasal colonization of neonatal rats. J Bacteriol 191:571–575. doi:10.1128/JB.00950-08. 19011027 PMC2620824

[38] Quintero B, Araque M, van der Gaast-de Jongh C, Escalona F, Correa M, Morillo-Puente S, Vielma S, Hermans PW 2011 Epidemiology of Streptococcus pneumoniae and Staphylococcus aureus colonization in healthy Venezuelan children. Eur J Clin Microbiol Infect Dis 30:7–19. doi:10.1007/s10096-010-1044-6. 20803226 PMC2998637

[39] Ebruke C, Dione MM, Walter B, Worwui A, Adegbola RA, Roca A, Antonio M 2016 High genetic diversity of Staphylococcus aureus strains colonising the nasopharynx of Gambian villagers before widespread use of pneumococcal conjugate vaccines. BMC Microbiol 16:38. doi:10.1186/s12866-016-0661-3. 26969294 PMC4788959

[40] Melles DC, Bogaert D, Gorkink RF, Peeters JK, Moorhouse MJ, Ott A, van Leeuwen WB, Simons G, Verbrugh HA, Hermans PW, van Belkum A 2007 Nasopharyngeal co-colonization with Staphylococcus aureus and Streptococcus pneumoniae in children is bacterial genotype independent. Microbiology 153:686–692. doi:10.1099/mic.0.2006/002279-0. 17322188

[41] Marks LR, Parameswaran GI, Hakansson AP 2012 Pneumococcal interactions with epithelial cells are crucial for optimal biofilm formation and colonization in vitro and in vivo. Infect Immun 80:2744–2760. doi:10.1128/IAI.00488-12. 22645283 PMC3434590

[42] Wang Z, Xiang Q, Yang T, Li L, Yang J, Li H, He Y, Zhang Y, Lu Q, Yu J 2016 Autoinducer-2 of Streptococcus mitis as a target molecule to inhibit pathogenic multi-species biofilm formation in vitro and in an endotracheal intubation rat model. Front Microbiol 7:88. doi:10.3389/fmicb.2016.00088. 26903968 PMC4744849

[43] Metersky ML, Masterton RG, Lode H, File TM Jr., Babinchak T 2012 Epidemiology, microbiology, and treatment considerations for bacterial pneumonia complicating influenza. Int J Infect Dis 16:e321–e331. doi:10.1016/j.ijid.2012.01.003. 22387143

[44] Chertow DS, Memoli MJ 2013 Bacterial coinfection in influenza: a grand rounds review. JAMA 309:275–282. doi:10.1001/jama.2012.194139. 23321766

[45] McCullers JA 2006 Insights into the interaction between influenza virus and pneumococcus. Clin Microbiol Rev 19:571–582. doi:10.1128/CMR.00058-05. 16847087 PMC1539103

[46] Joseph C, Togawa Y, Shindo N 2013 Bacterial and viral infections associated with influenza. Influenza Other Respir Viruses 7(Suppl 2):105–113. doi:10.1111/irv.12089. 24034494 PMC5909385

[47] Guiral S, Mitchell TJ, Martin B, Claverys JP 2005 Competence-programmed predation of noncompetent cells in the human pathogen Streptococcus pneumoniae: genetic requirements. Proc Natl Acad Sci U S A 102:8710–8715. doi:10.1073/pnas.0500879102. 15928084 PMC1150823

[48] Ghoul M, West SA, Johansen HK, Molin S, Harrison OB, Maiden MC, Jelsbak L, Bruce JB, Griffin AS 2015 Bacteriocin-mediated competition in cystic fibrosis lung infections. Proc Biol Sci 282. doi:10.1098/rspb.2015.0972. PMC4571691 26311664

[49] Majeed H, Lampert A, Ghazaryan L, Gillor O 2013 The weak shall inherit: bacteriocin-mediated interactions in bacterial populations. PLoS One 8:e63837. doi:10.1371/journal.pone.0063837. 23704942 PMC3660564

[50] Selva L, Viana D, Regev-Yochay G, Trzcinski K, Corpa JM, Lasa I, Novick RP, Penadés JR 2009 Killing niche competitors by remote-control bacteriophage induction. Proc Natl Acad Sci U S A 106:1234–1238. doi:10.1073/pnas.0809600106. 19141630 PMC2633583

[51] Lister JL, Horswill AR 2014 Staphylococcus aureus biofilms: recent developments in biofilm dispersal. Front Cell Infect Microbiol 4:178. doi:10.3389/fcimb.2014.00178. 25566513 PMC4275032

[52] Peschel A, Otto M 2013 Phenol-soluble modulins and staphylococcal infection. Nat Rev Microbiol 11:667–673. doi:10.1038/nrmicro3110. 24018382 PMC4780437

[53] van de Rijn I, Kessler RE 1980 Growth characteristics of group A streptococci in a new chemically defined medium. Infect Immun 27:444–448. 6991416 10.1128/iai.27.2.444-448.1980 PMC550785

[54] van Schilfgaarde M, van Alphen L, Eijk P, Everts V, Dankert J 1995 Paracytosis of Haemophilus influenzae through cell layers of NCI-H292 lung epithelial cells. Infect Immun 63:4729–4737. 7591129 10.1128/iai.63.12.4729-4737.1995 PMC173678

[55] Andersson B, Dahmén J, Frejd T, Leffler H, Magnusson G, Noori G, Edén CS 1983 Identification of an active disaccharide unit of a glycoconjugate receptor for pneumococci attaching to human pharyngeal epithelial cells. J Exp Med 158:559–570. doi:10.1084/jem.158.2.559. 6886624 PMC2187347

[56] Avery OT, Macleod CM, McCarty M 1944 Studies on the chemical nature of the substance inducing transformation of pneumococcal types: induction of transformation by s desoxyribonucleic acid fraction isolated from pneumococcus type III. J Exp Med 79:137–158. doi:10.1084/jem.79.2.137. 19871359 PMC2135445

[57] Gillaspy AF, Hickmon SG, Skinner RA, Thomas JR, Nelson CL, Smeltzer MS 1995 Role of the accessory gene regulator (agr) in pathogenesis of staphylococcal osteomyelitis. Infect Immun 63:3373–3380. 7642265 10.1128/iai.63.9.3373-3380.1995 PMC173464

[58] Marks LR, Clementi EA, Hakansson AP 2013 Sensitization of Staphylococcus aureus to methicillin and other antibiotics in vitro and in vivo in the presence of HAMLET. PLoS One 8:e63158. doi:10.1371/journal.pone.0063158. 23650551 PMC3641093

[59] Kjos M, Aprianto R, Fernandes VE, Andrew PW, van Strijp JA, Nijland R, Veening JW 2015 Bright fluorescent Streptococcus pneumoniae for live-cell imaging of host-pathogen interactions. J Bacteriol 197:807–818. doi:10.1128/JB.02221-14. 25512311 PMC4325099

[60] Bose JL, Fey PD, Bayles KW 2013 Genetic tools to enhance the study of gene function and regulation in Staphylococcus aureus. Appl Environ Microbiol 79:2218–2224. doi:10.1128/AEM.00136-13. 23354696 PMC3623228

[61] Tait AR, Davidson BA, Johnson KJ, Remick DG, Knight PR 1993 Halothane inhibits the intraalveolar recruitment of neutrophils, lymphocytes, and macrophages in response to influenza virus infection in mice. Anesth Analg 76:1106–1113. doi:10.1213/00000539-199305000-00033. 8484515

[62] Tyx RE, Roche-Hakansson H, Hakansson AP 2011 Role of dihydrolipoamide dehydrogenase in regulation of raffinose transport in Streptococcus pneumoniae. J Bacteriol 193:3512–3524. doi:10.1128/JB.01410-10. 21602335 PMC3133304

